# Supplementary material for: Evidence supported by Mendelian randomization: impact on inflammatory factors in knee osteoarthritis
Source: Front Med (Lausanne). 2024 May 28;11:1382836. doi: 10.3389/fmed.2024.1382836 (PMC11165061; doi:10.3389/fmed.2024.1382836)
Supplement: Supplementary file 1 [file Data_Sheet_1.ZIP › data/GCST90274838_5e6/838.leaveoneout.pdf]

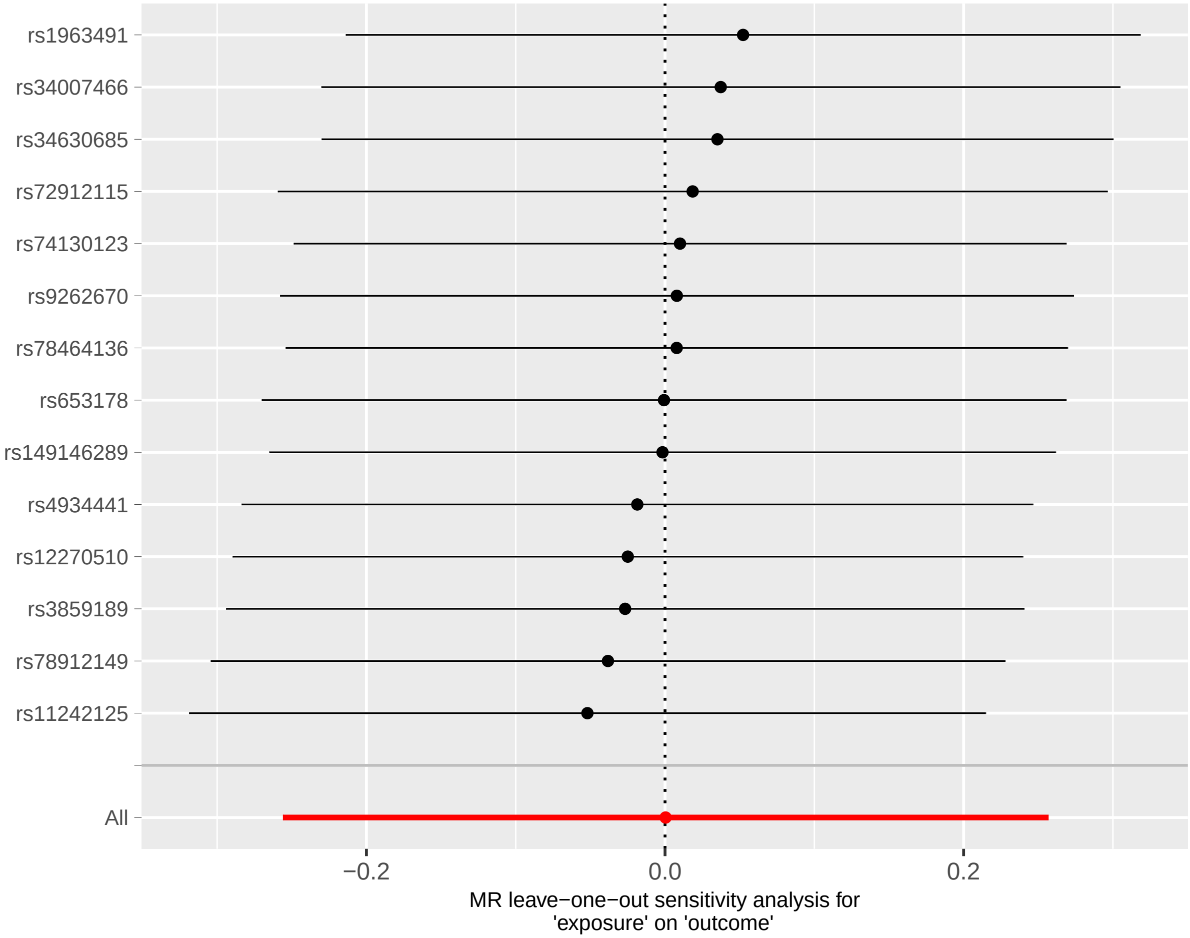
rs1963491
rs34007466
rs34630685
rs72912115
rs74130123
rs9262670
rs78464136
rs653178
rs149146289
rs4934441
rs12270510
rs3859189
rs78912149
rs11242125
All
−0.2
0.0
0.2
MR leave−one−out sensitivity analysis for
'exposure' on 'outcome'
